# Ammonium Transport Proteins with Changes in One of the Conserved Pore Histidines Have Different Performance in Ammonia and Methylamine Conduction

**DOI:** 10.1371/journal.pone.0062745

**Published:** 2013-05-07

**Authors:** Jinan Wang, Tim Fulford, Qiang Shao, Arnaud Javelle, Huaiyu Yang, Weiliang Zhu, Mike Merrick

**Affiliations:** 1 Drug Discovery and Design Center, CAS Key Laboratory of Receptor Research, Shanghai Institute of Materia Medica, Chinese Academy of Sciences, Shanghai, China; 2 Department of Molecular Microbiology, John Innes Centre, Norwich Research Park, Norwich, United Kingdom; University of Cambridge, United Kingdom

## Abstract

Two conserved histidine residues are located near the mid-point of the conduction channel of ammonium transport proteins. The role of these histidines in ammonia and methylamine transport was evaluated by using a combination of *in vivo* studies, molecular dynamics (MD) simulation, and potential of mean force (PMF) calculations. Our *in vivo* results showed that a single change of either of the conserved histidines to alanine leads to the failure to transport methylamine but still facilitates good growth on ammonia, whereas double histidine variants completely lose their ability to transport both methylamine and ammonia. Molecular dynamics simulations indicated the molecular basis of the *in vivo* observations. They clearly showed that a single histidine variant (H168A or H318A) of AmtB confines the rather hydrophobic methylamine more strongly than ammonia around the mutated sites, resulting in dysfunction in conducting the former but not the latter molecule. PMF calculations further revealed that the single histidine variants form a potential energy well of up to 6 kcal/mol for methylamine, impairing conduction of this substrate. Unlike the single histidine variants, the double histidine variant, H168A/H318A, of AmtB was found to lose its unidirectional property of transporting both ammonia and methylamine. This could be attributed to a greatly increased frequency of opening of the entrance gate formed by F215 and F107, in this variant compared to wild-type, with a resultant lowering of the energy barrier for substrate to return to the periplasm.

## Introduction

Ammonium transport is facilitated by a highly conserved family of membrane proteins, represented by the ammonium transport (Amt) proteins in bacteria,[1]–[4] plants,[5]–[7] and yeast (where they are designated methylamine permease or Mep proteins), [8], [9] and by the Rhesus (Rh) proteins in animals. [10], [11] High resolution structures have been determined for *Escherichia coli* AmtB (EcAmtB), [12], [13] Amt-1 from *Archaeoglobus fulgidus*, [14] Rh50 from *Nitrosomonas europaea*, [15], [16] and human RhCG, [17] all of which show considerable structural conservation. All the proteins are homotrimers in which each subunit contains a highly hydrophobic substrate-conducting pore. These characteristics of the pore make it favorable for conduction of electroneutral species (e.g. NH_3_) rather than ions (e.g. NH_4_^+^), although extracellular ammonia should exist predominantly in the positively charged form under normal physiological conditions.

Despite the availability of a number of structures and a variety of analyses, both biochemically and computationally, the mechanism of ammonium conduction remains controversial. [18], [19] To date, there are at least four suggested conduction mechanisms, namely electroneutral NH_3_ transport,[12], [13], [20]–[24] NH_3_/H^+^ symport,[14], [25]–[27] NH_4_^+^ transport, [28], [29] and the antiport of NH_4_^+^/H^+^. [30], [31] Computational simulations have focussed on EcAmtB and have predominantly supported the electroneutral NH_3_ transport model. These simulations also suggested that prior to electroneutral ammonia being transported into the cytoplasm, an NH_4_^+^ ion is bound in the extracytoplasmic vestibule and is subsequently deprotonated by a mechanism that is still a matter of debate.[25], [32]–[43].

As mentioned above, the pore of EcAmtB is lined with hydrophobic residues, and there is a pair of histidines, His168 and His318, near the mid-point. These two histidines are highly conserved in both the Amt and Rh families, and they have been postulated to play a critical role in mediating ammonia transport. [12], [13], [44] Using mutant analysis and methylamine (a general analogue of ammonia in experiment) transport assays, the role of these histidines in substrate conductance by EcAmtB was investigated by us previously. [44] We analyzed 14 engineered polar and non-polar variants, and showed that all the variants, with the exception of H168E, were ineffective in methylamine transport and hence by inference also for transport of ammonium. [44] Substitution of the first His residue within the Amt channel by a glutamate is a natural variant seen in a number of fungal Amt proteins, [44] suggesting that an acidic residue may be able to substitute for the function of the histidine, at least in some cases.

The structures of six EcAmtB variants (H168A, H168E, H168F, H318A, H318F and H168A/H318A) were also determined in our earlier study. [44] Compared with wild-type EcAmtB (1U7G), [13] the structural changes were restricted to the mutated histidine residue with occasional minor conformational changes in the neighbouring histidine. [44] The structural insensitivity of EcAmtB toward histidine mutation was further illustrated by the superposition of the Cα positions of these structures (Cα_RMSD <0.4 Å). [44].

These observations raise a number of questions. What is the exact role of the two histidine residues at the atomic level? Why are the histidine variants of EcAmtB unable to transport methylamine, and do they indeed have the same phenotype with respect to ammonia? This last question raises an important issue that has received relatively little attention in experimental studies on Amt proteins: namely, is it reasonable to use methylamine as an ammonium mimic and do both molecules behavior similarly in these proteins?

To address these questions, a combined experimental and molecular dynamics (MD) simulation study was perform to investigate the behaviors of the endogenous substrate (ammonium) and its analogue [^14^C] methylammonium (MA) in a wild-type Amt protein and its His variants. The potential of both EcAmtB and *Saccharomyces cerevisiae* Mep2 (ScMep2) proteins carrying alterations in the conserved His residues to support growth on ammonium as sole nitrogen source was analyzed and these experiments indicated that, unlike when methylamine is the substrate, removal of a single conserved His residue does not impair growth on ammonia but the double His variant does. MD simulations and potential of mean force (PMF) calculations on wild-type EcAmtB and the His variants were then conducted to study the detailed mechanism of the molecular interactions between AmtB and the substrates. Both the *in vivo* experiments and the simulations indicated that His variants of AmtB do indeed have different capabilities in conducting ammonium and methylamine. The simulations revealed that dysfunction of a single His variant in conducting methylamine could be attributed to the deep potential energy well around the mutated site that traps the substrate and impairs translocation. By contrast, the double His variant significantly changes the intrinsic vibration mode of the entrance gate, leading to the loss of the unidirectional property of AmtB as an ammonia conductor.

## Materials and Methods

### Plasmid Construction

Previously constructed mutant alleles of *E. coli AmtB* [23], [44] were amplified by PCR to introduce *Xho*I and *Bam*HI sites at the 5′ and 3′ ends respectively and then cloned into plasmid pDR195 which allows constitutive expression from the yeast ATPase promoter. [45] Similar plasmids were constructed expressing wild-type AmtB, and the variants H168A, H318A and H168A/H318A (Table 1). A comparable set of mutants of the *S. cerevisiae mep2* gene were also constructed (Table 1). Wild-type *mep2* was amplified from chromosomal DNA of strain 23344c [8] and cloned into pDR195 in a comparable manner to *E. coli amtB,* giving plasmid pTF28. The conserved His residues in Mep2 are H194 and H348. Mutant alleles of these residues encoding H194A and H348A were derived using the QuikChange mutagenesis kit (Stratagene). These individual mutations were then also combined to give a double His variant, H194A/H348A. The expressed proteins in *S. cerevisiae* strains were detected with anti-EcAmtB antibody by Western blotting as described previously. [46].

**Table 1:** Strains and plasmids.

	Relevant Genotype	Reference
**Strain**		
***S. cerevisiae***		
23344c	*MAT*α *ura3*	[8]
31019b	*MAT*α *ura3 mep1Δ* *mep2* *Δ::LEU2 mep3 Δ::KanMX2*	[8]
**Plasmid**		
pDR195	*E. coli-S. cerevisiae* shuttle vector	[45]
pTF14	*E. coli amtB* in pDR195	This work
pTF17	*E. coli amtB* H168A in pDR195	This work
pTF18	*E. coli amtB* F215A in pDR195	This work
pTF19	*E. coli amtB* H318A in pDR195	This work
pTF20	*E. coli amtB* H168A, H318A in pDR195	This work
pTF28	*S. cerevisiae mep2* in pDR195	This work
pTF29	*S. cerevisiae mep2* H194A in pDR195	This work
pTF31	*S. cerevisiae mep2* H348A in pDR195	This work
pTF32	*S. cerevisiae mep2* H194A H3489A in pDR195	This work

### Assessment of Methylamine Uptake Using [^14^C] Methylammonium

The methylamine uptake ability of the various Amt proteins was assessed by transferring each plasmid construct into *S. cerevisiae* strain 31019b that lacks all three wild-type *mep* genes. [8] Methylamine uptake was assessed using [^14^C] methylammonium as previously described. [47] Data are the average of three biological replicates.

### Assessment of Ammonia Uptake by Growth on Ammonium

The ammonia-uptake phenotype of each of the AmtB and Mep2 variants was assessed in *S. cerevisiae*. Cultures were grown overnight at 30°C on YNB glutamate medium as described previously. [47] Cells were then washed and resuspended to an OD_600_ of 0.3 in 10 mM phosphate buffer pH 6.0 containing 3% (wt/vol) glucose. A 5 µl aliquot was spotted onto agar plates of YNB medium (pH 6.0) containing 3 mM NH_4_Cl and growth was visualised after 5 days incubation at 30°C. Growth rates were also measured in liquid medium to compare growth on ammonium with a control nitrogen source, namely glutamate. Cultures were pre-grown in YNB with either glutamate (30 mM) or NH_4_Cl (3 mM) for 24 h at 30°C. Cells were washed in 10 mM phosphate buffer pH 6.0 containing 3% (wt/vol) glucose, and then resuspended at an OD_600_ of 0.3 in 10 ml of YNB plus either glutamate (30 mM) or NH_4_Cl (3 mM). These cultures were incubated at 30°C shaking at 220 rpm and the growth rate in log phase was determined. Data are the means from six replicate experiments.

### Molecular Dynamics Simulations

**Figure 1:**
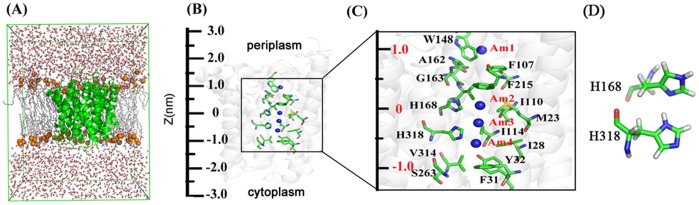
The system used for MD simulations. *A*, Side view of the simulation system. AmtB is shown with the green-colored ribbon representation. Phosphate atoms of the lipid are drawn as orange spheres and the other atoms are represented as white lines (hydrogen atoms are not shown for clarity). Water molecules are displayed as red and white sticks. The front half of the bilayer is not shown for clarity. *B*, The structure of the AmtB channel (1U7G) with z axis indicated on the left. *C*, The detailed structure from the Am1 site to Am4 site in the AmtB channel (from z = −1.5 nm to z = 1.5 nm). Certain key residues are indicated to facilitate the interpretation of the AmtB PMFs (Figs. 4*A* and 4*B*). D, The state of the neutral His-168–His-318 system used in the simulation.

The computational approach for MD simulations was similar to our previous work. [33], [36] In brief, the x-ray structure of EcAmtB (PDB entry 1U7G, Figure 1) determined by Khademi et al [13] was used as the initial structure (many previous studies indicated that the use of AmtB monomer [33], [35], [36], [43] generates the similar results as using trimer, [32], [39]–[41] so the present simulation used the monomer instead of trimer as the initial structure). The protein was fitted into the dipalmitoylphosphatidylcholine (DPPC) bilayer with 181 lipids (23530 atoms) and solvated in a bath of 13137 TIP3P water molecules [48] to generate a suitable membrane system. Four possible locations for substrate molecules were designated as Am1-4. [13] The substrate, viz. CH_3_NH_2_ or NH_3_, was manually added to the Am2 sites (Figure 1B). Na^+^/Cl^−^ ions were then added to neutralize the modeling system. The same protonation state of His168-His318 as our previous study (shown in Fig. 1D), [33], [36] was used in all simulations. In total, 12 simulation systems were designed (Table 2).

**Table 2:** Summary of MD simulations on wild type AmtB and His variants, and deduced conductivity compared to that observed *in vivo*.

	Protein	Substrate	*t*_simulation_ (ns)	*t*_exiting_ (ns)	Conductivity (fromsimulation)	Conductivity (fromexperiment)
A1	wild-type	CH_3_NH_2_	20	13.950	Conductive	Conductive
A2	wild-type	NH_3_	20	1.792	Conductive	Conductive
B1	H168A	CH_3_NH_2_	100	–	Nonconductive	Nonconductive
B2	H168A	NH_3_	20	4.415	Conductive	Conductive
C1	H318A	CH_3_NH_2_	100	–	Nonconductive	Nonconductive
C2	H318A	NH_3_	20	8.860	Conductive	Conductive
D1	H168A/H318A	CH_3_NH_2_	20	5.190	Exit to periplasm	Nonconductive
D2	H168A/H318A	NH_3_	40	37.460	Exit to periplasm	Nonconductive
D3	H168A/H318A	CH_3_NH_2_	100	3.700	Exit to cytoplasm	Nonconductive
D4	H168A/H318A	NH_3_	100	78.420	Exit to cytoplasm	Nonconductive
D5	H168A/H318A	–	20			
D6	H168A/H318A	–	20			

The initial location of the substrate is always at site Am2. Simulations D5 and D6 both without substrate are designed to explore the intrinsic dynamic property of the conformations of the Phe gate (F107 and F215).

All MD simulations were performed using GROMACS 4.5.3 [49] with the charmm27 force fields. [50], [51] It is worth noting that in the simulations, both ammonia and methylamine took the neutral form. There are actually lots of theoretical researches showing that it should be the neutral form but not the charged form for the two substrates in the pore of the AmtB. For example, Bostick et al. [39] calculated the pKa (NH_3_/NH_4_^+^) profile along the channel and found an apparent pKa shifted upward by ∼5 units only happening at site Am1 and near the exit site (around S263) of the channel. Ishikita et al [37] also calculated the pKa (NH_3_/NH_4_^+^) in the four binding sites (Am1-4) and the results also showed that the ammonia should be depronated at site Am1. The force field parameters of ammonia and methylamine were obtained from server SwissParam. [52] In order to validate the force field parameters, the salvation free energy of ammonia and methylamine in water was calculated using free energy perturbation (detailed protocol shown in Text S1). The calculated solvation free energies for ammonia and methylamine are −5.00±0.24 and −4.49±0.18 kcal/mol (Figure S1) respectively, very similar to the experiment values (−4.31 kcal/mol for ammonia and −4.57 kcal/mol for methylamine [53]). Therefore the parameters used here should be suitable for simulations.

During the MD simulation, all the bonds including hydrogen atoms were constrained with the linear constraint solver algorithm [54] and the integration step of 2 fs was used. Electrostatic interactions were calculated using the particle-mesh Ewald method. [55] The cutoff for Lennard-Jones interactions was set as 12 Å. The temperature was kept constant at 323 K by coupling the system to a thermal bath recently developed by Bussi et al [56] with a coupling time of 0.1 ps. A constant pressure of 1.0 bar with Berendsen bath [57] was applied independently in X, Y and Z directions of the system with a coupling constant of 1.0 ps.

Each simulation system was subjected to energy minimizations using the steepest- descents algorithm. Then, a 250 ps MD simulation was carried out to heat the system to 323 K with the protein, lipid and the substrate fixed, followed by another 250 ps MD simulation with the protein main chain, the phosphorus atoms of the lipid, and substrate fixed. After that, the whole system was relaxed except for protein C_α_ atoms and substrate for 10 ns MD simulation. Based on the relaxed system, the conventional MD simulation was performed without any constraints for 100 ns or stopped once the substrate left the channel.

### Umbrella Sampling Simulations

The starting frames for the umbrella sampling were taken each from the first 10 ns equilibrium simulations. The sampling with the box length fixed in the z direction was carried out by applying a harmonic restraint force along the pore coordinate with a force constant of 800 kJ/mol/nm^2^ on the heavy atoms of the substrates. The EcAmtB channels were divided into 0.15 Å wide equidistant sections parallel to the membrane with the center of each section representing an umbrella center. The simulation temperature was kept constant at 300 K by coupling the system to a Nosé-Hoover thermostat [58], [59] (τ = 0.5 ps). Likewise, the pressure was kept at 1 bar using the Parrinello- Rahman [60] pressure coupling scheme (τ = 1 ps). The cutoff for Lennard-Jones interaction was set as 10 Å. To enhance sampling, a similar protocol to that described by Hub et al. [61] was employed by keeping a distance of at least 15 Å between the solutes. After energy minimization, 100 ps MD simulation was carried out to heat and equilibrate the system to 300 K with the protein and the substrate fixed, then each umbrella simulation was carried out for 500 ps. After removing the first 150 ps for equilibration, umbrella histograms were extracted from the z-coordinate of the restrained atom. The PMFs were computed using a periodic implementation of the weighted histogram analysis method. [62], [63] All the free energy profiles were calculated with the g wham program [63] in GROMACS 4.5.3.

## Results

### *In Vivo* Effects of Changing the Conserved His Residues on Substrate Conduction in AmtB

In our previous *in vivo* studies of the role of the conserved twin-His motif we used the ammonium analogue [^14^C] MA instead of ammonium. We demonstrated that both histidines are essential for transport of MA and hence by inference also for transport of ammonium. [44] To assess ammonium-dependent growth in this study we took advantage of the very clear ammonium-dependent growth phenotype of *S. cerevisiae* lacking all three *amt* (*mep*) genes. [8] This phenotype can be complemented by heterologous expression of Amt proteins (or the closely related Rhesus proteins) from a variety of organisms. [44], [47], [64] So we expressed wild-type EcAmtB and the variants H168A, H318A, H168A/H318A in *S. cerevisiae* strain 31019b. We confirmed that all the EcAmtB variant proteins were present in the *S. cerevisiae* cell membrane by Western blotting of membrane fractions using an anti-EcAmtB antibody (data not shown).

**Figure 2:**
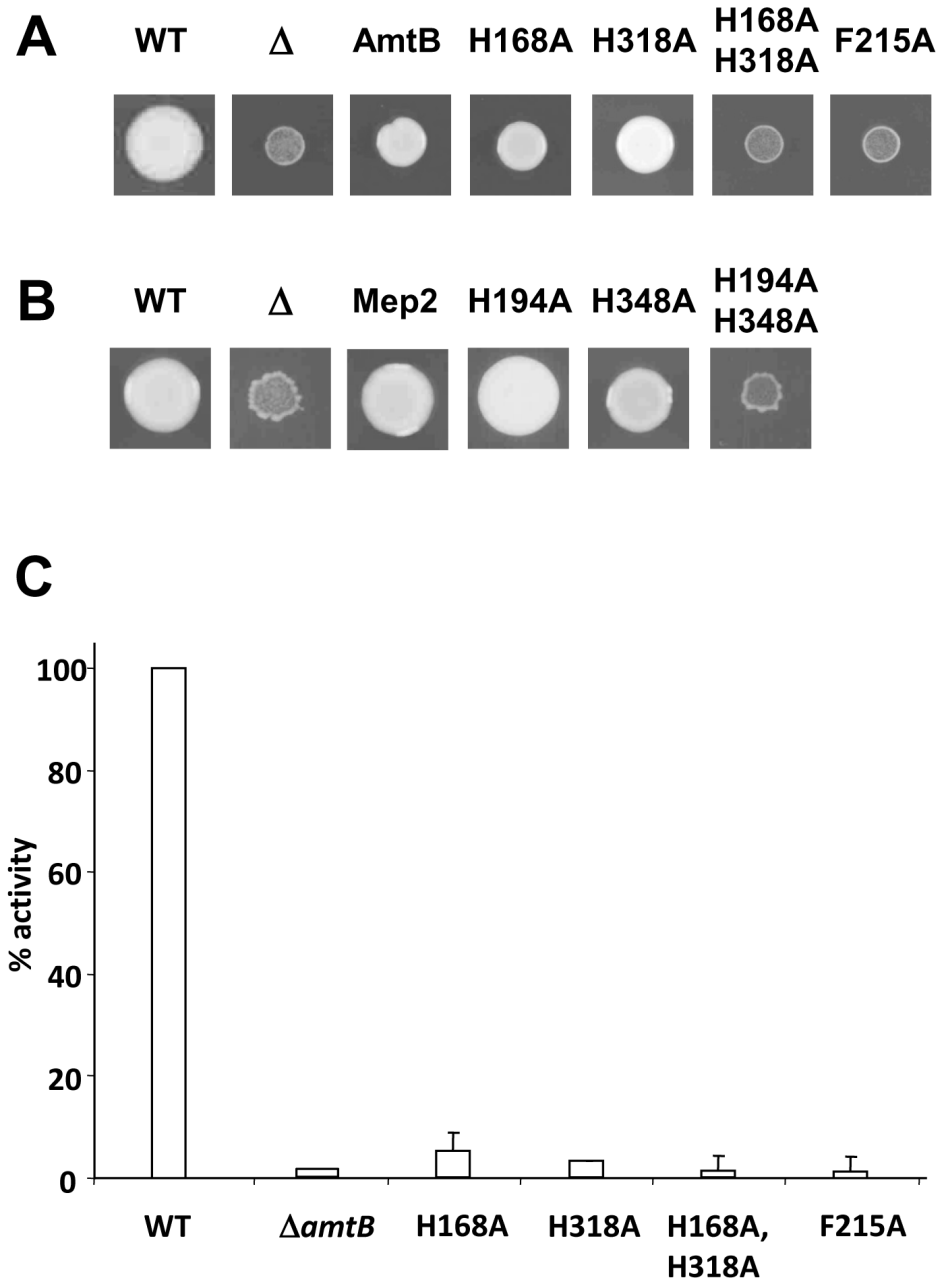
Ammonium-dependent growth of AmtB and Mep2 His variants. *A*, Growth of *S. cerevisae* strains after 5 days at 30°C on YNB medium pH 6.0 containing 3 mM NH_4_Cl. WT - *S. cerevisiae* strain 23344c, Δ-*S. cerevisiae* strain 31019b lacking all three *mep* genes, AmtB - strain 31019b expressing wild-type *E. coli* AmtB, or with mutated versions of *amtB* changing the residues indicated – final four lanes. *B*, as for *A*, but with *S. cerevisiae* strain 31019b now expressing Mep2 or mutated versions of *mep2* changing the residues indicated – final three lanes. *C*, Methylammonium uptake activity of wild-type *E. coli* AmtB and variants, measured in *E. coli* (Data taken from Ref [23], [44]).

Measurements of [^14^C] MA transport by these *S. cerevisiae* strains (data not shown) mirrored closely those reported previously for *E. coli.* ^26,45^ Variants H168A, H318A, and the double H168A/H318A all showed essentially no transport. Surprisingly, the phenotypes of the EcAmtB His variants with respect to their abilities to support ammonium-dependent growth in *S. cerevisiae* differed markedly from those observed for MA transport (Figures 2A and 2C). As expected, the *S. cerevisiae Δmep* strain showed almost no growth after 5 days at 30°C on 3 mM NH_4_Cl. However, the two single His variants (H168A and H318A) grew as well as the strain expressing wild-type EcAmtB and only the H168A/H318A double mutant failed to grow.

To exclude the possibility that the phenotypes of the His variants are artefacts due to heterologous expression of EcAmtB in *S. cerevisiae*, we constructed an equivalent set of variants in ScMep2 expressed from the same plasmid. Unlike both *S. cerevisiae* Mep1 and Mep3, in which the first His residue in the conduction channel (equivalent to H194) is replaced by a glutamate residue, the ScMep2 protein contains both conserved His residues (H194 and H348). We therefore constructed the two single variants (H194A and H384A) and the double variant (H194A/H384A) of ScMep2, and we assessed [^14^C] MA transport and ammonium-dependent growth for the *S. cerevisiae Δmep* strain expressing wild-type ScMep2 and each variant. [^14^C] MA conduction was completely impaired in all three His variants each of which showed no significant [^14^C] MA uptake when compared with the *mep* strain (data not shown). In marked contrast, growth on NH_4_Cl showed the same pattern as observed with EcAmtB, namely the growth of the single His variants was unimpaired whereas the double mutant failed to grow (Figure 2B).

To confirm that these effects of the particular variants are specific to growth on ammonium and do not reflect a general growth impairment, we measured growth rates in liquid medium for all of the above-mentioned variants of either EcAmtB or ScMep2 expressed in the *S. cerevisiae Δmep* strain, using either 3mM NH_4_Cl or 30 mM glutamate as sole N-source. The data revealed no very marked differences between any of the EcAmtB or ScMep2 variants when grown on glutamate, but a very marked effect for the double His variants (AmtB H168A/H318A and Mep2 H194A/H384A) and for EcAmtB F215A in ammonium (Table 3). Hence it is clear that the His double mutants of both EcAmtB and ScMep2 do not have a general growth defect and that their very distinctive phenotypes on ammonium are likely to reflect the abilities of these Amt channel variants to conduct that substrate.

**Table 3:** Growth characteristics of EcAmtB and ScMep2 variants.

Amt phenotype	Growth rate (OD_600_/hr)	
	Glutamate	NH_4_Cl
*S. cerevisae* wild-type	0.175±0.005	0.150±0.007
*S. cerevisae Δmep*	0.175±0.007	0.015±0.004
EcAmtB wild-type	0.120±0.006	0.080±0.005
EcAmtB H168A	0.145±0.008	0.080±0.007
EcAmtB H318A	0.148±0.008	0.060±0.019
EcAmtB H168A/H318A	0.145±0.010	0.015±0.001
EcAmtB F215A	0.140±0.008	0.010±0.001
ScMep2 wild-type	0.135±0.005	0.115±0.007
ScMep2 H194A	0.165±0.012	0.090±0.005
ScMep2 H348A	0.145±0.014	0.100±0.010
ScMep2 H194A/H348A	0.135±0.010	0.015±0.002

*S. cerevisae* strains were grown in YNB liquid medium pH 6.0 with either 30 mM glutamate or 3 mM NH_4_Cl as the sole nitrogen source. The growth rate in logarithmic phase was recorded as OD_600_/hr and the data are the means of six replicate experiments. Wild-type *S. cerevisae* (strain 23344c) and *S. cerevisae Δmep* strain (31019b) were used as controls. EcAmtB and ScMep2 variants were all expressed in *S. cerevisae Δmep* strain (31019b).

In summary, these *in vivo* data showed that changing just one of the conserved His residues in the Amt conduction channel does not prevent transport of ammonia but, as previously reported, [44] does impair transport of the slightly larger ammonia analogue [^14^C] methylamine. Only variants lacking both His residues failed to transport ammonia. We therefore endeavoured to use MD simulations and PMF calculations to study the different behaviours of ammonia and methylamine in the His-mutated EcAmtB conduction channel and thereby to investigate the mechanism(s) underlying these behaviours.

### Substrate Conduction through Wild-type EcAmtB

**Figure 3:**
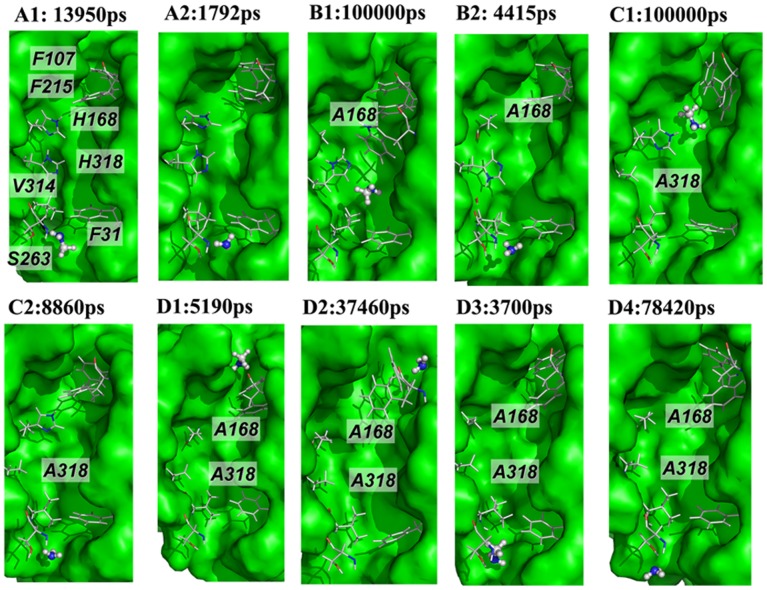
Process of substrate leaving the channel in wild-type and His mutants of AmtB. *A1*, wild-type AmtB with CH_3_NH_2_ (13950 ps from trajectory A1). *A2*, wild-type AmtB with NH_3_ (1792 ps from trajectory *A2*). *B1*, mutant H168A with CH_3_NH_2_ (100 ns from trajectory *B1*). *B2*, mutant H168A with NH_3_ (4415 ps from trajectory B2 ). *C1*, mutant H318A with CH_3_NH_2_ (100 ns from trajectory *C1*). *C2*, mutant H318A with NH_3_ (8860 ps from trajectory *C2*). *D1*, mutant H168A/H318A with CH_3_NH_2_ (5190 ps from trajectory *D1*). *D2*, mutant H168A/H318A with NH_3_ (37460 ps from trajectory *D2*). *D3*, mutant H168A/H318A with CH_3_NH_2_ (3700 ps from trajectory *D3*). *D4*, mutant H168A/H318A with NH_3_ (78420 ps from trajectory *D4*).

The MD simulations showed that methylamine and ammonia could be transported by the channel at around 14 ns and 1.8 ns, respectively (Figures 3 A1 & A2), consistent with our previous MD simulation [33] and the present *in vivo* experimental results (Figure 2). Moreover, similar to our previous reports, [33] five residues, viz., H168, H318, Y32, S263, and I110, were found to be important for substrate translocation by forming hydrogen bonds frequently with both substrates (calculated by the program LIGPLOT4.4.2 [65], Figure S1B and S1C). However, frequent hydrophobic interactions with the hydrophobic residues in the channel were observed during methylamine conduction, indicating a more complex conduction mechanism for methylamine than for ammonia (Figure S2A).

**Figure 4:**
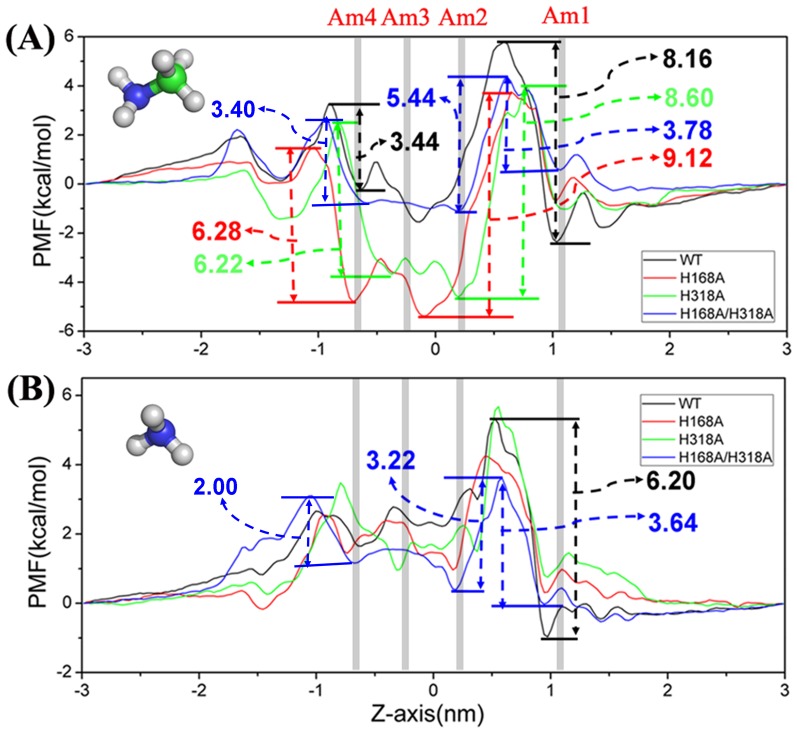
PMFs for substrate permeation across wild-type and His variants of AmtB. Permeation of *A*, CH_3_NH_2_ and *B*, NH_3_ across wild-type, H168A, H318A, and H168A/H318A AmtB (black, red, green and blue curves, respectively).

PMF results revealed that the largest energy barrier from site Am1 (z ≈1.0 nm) to Am2 (z≈0 nm) is about 8.16 and 6.20 kcal/mol for methylamine and ammonia translocation, respectively (Figures 4A & B), corresponding to the entrance of the substrates into the channel (Figure 1). Once the substrates pass the energy barrier they can move easily along the channel to the cytoplasmic side, only having to overcome a small energy barrier along the rest pathway from z≈0 to −1 nm (Figure 1). In addition, the PMF value for ammonia is always positive, indicating that it may spontaneously move downhill to the exit. Therefore, ammonia should be conducted easily (∼1.8 ns observed in trajectory A2). However, the PMF value for methylamine is as low as −1.53 kcal/mol around site Am2, which is at least partially attributable to the hydrophobic interaction between methylamine and aromatic residue W212. The energy barriers around the exit gate are about 1.0 and 3.44 kcal/mol for ammonia and methylamine respectively, which is in well agreement with the reduced conductivity of methylamine compared to ammonia. On the other hand, the key residues involved in hydrogen-bonding for conduction of the two substrates are the same, namely H168, H318, Y32 and S263. Therefore, although there are some differences in the detailed PMF values, the overall performances of ammonia and methylamine in wild-type EcAmtB are rather similar, confirming that methylamine should be an appropriate analogue of ammonia to investigate the function of the wild-type EcAmtB channel.

### Substrate Conduction through the H168A Variant of EcAmtB

**Figure 5:**
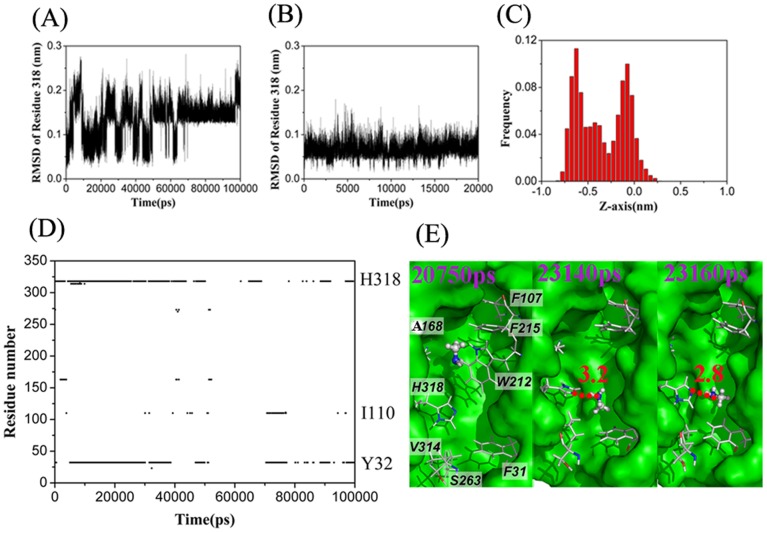
Effects of methylamine conduction through H168A AmtB. The root-mean-square deviations (RMSD) of the atoms of His318 relative to their initial structure as a function of simulation time from the trajectories of methylamine (*A*) and ammonia (*B*) through H168A AmtB. *C*, Frequency distribution for the location of methylamine along the Z-axis of the channel in H168A AmtB (trajectory B1, data were collected in the whole trajectory). D, The residues of H168A AmtB involved in hydrogen bonds with methylamine versus simulation time in the trajectory B1. *E*, Side views of three snapshots (at 20750, 23140, and 23160 ps, respectively) showing the trap of methylamine at site z≈0 nm by forming hydrophobic contacts with residues around A168 and at z≈−0.6 nm by forming a hydrogen bond with H318 respectively. Hydrogen bonds are shown as red dashed lines.

As shown in Figures 3B1 & 3B2, ammonia could easily transit the H168A variant within 5 ns of simulation time whereas methylamine could not within 100 ns. Comparing the two trajectories of B1 and B2, we found that residue H318 showed more fluctuation in the presence of methylamine than of ammonia (Figures 5A & B). This greater fluctuation of H318 is likely to be induced by its stable hydrogen bonding to methylamine which was almost always observed in the simulation trajectory (Figures 5D & E). Moreover, there were also hydrophobic interactions between methylamine and residues around A168 (Figure 5E). As a result, methylamine was trapped around two sites (z≈−0.6 and 0 nm) (Figure 5C). By contrast, ammonia readily formed an NH-π interaction with residue W212, which facilitated the ammonia moving to site Am4. The necessity for the presence of an aromatic ring at residue 212 was demonstrated in our earlier studies, where we showed that a W212F variant of EcAmtB is active but a W212A variant is not. [23], [33] Once at site Am4, ammonia could easily exit from H168A EcAmtB.

The PMF profile for methylamine in the channel of the H168A variant has a different shape compared to that in wild-type EcAmtB: a deep potential energy well, which is about 9.12 kcal/mol and 6.28 kcal/mol lower than the entrance and exit sites respectively, exists in the range of z≈−0.8 nm to z≈0.7 nm. Within this range, two local energy minima, viz., −4.79 and −5.37 kcal/mol, are present at two sites (z≈−0.70 nm and −0.10 nm), corresponding to the positions where the methylamine was trapped in the MD simulation (Figure 5C). On the other hand, the PMF profile for ammonia transport in the channel of the H168A variant is similar to that of ammonia in wild-type EcAmtB, except that the energy barrier from site Am1 to Am2 is smaller (Figure 4B). The deep potential energy well in the PMF of methylamine but not ammonia in the channel of the H168A variant may partially be attributed to the more hydrophobic environment of the inner wall of the H168A variant, which is more sensitive for the hydrophobic methylamine but not for ammonia. Moreover, the free-energy perturbation was used to calculate the free energy difference between methylamine and ammonia at the site Am2 of H168A variant and in water solution respectively. The calculated value in the former case (2.4±0.50 kcal/mol) is apparently larger than that in the latter case (−0.51±0.42 kcal/mol, see Figure S1), indicating a large difference between the two substrates in the hydrophobic environment (e.g., in the channel) but not in water solution which to some extent validates the results of PMF calculation. The error bar for all PMF profiles was calculated and the data was quite small compared to the detailed free energy values (see Figure S3), which also suggests that the PMF calculation is reasonable.

### Substrate Conduction through the H318A Variant of EcAmtB

Two more MD simulations, C1 and C2, were performed to analyze the conduction of methylamine and ammonia in the channel of the H318A EcAmtB. As shown in Figures 3C1 & C2, the methylamine molecule was also trapped in the channel during the 100 ns simulation time whereas ammonia could again easily transit the channel within 10 ns.

**Figure 6:**
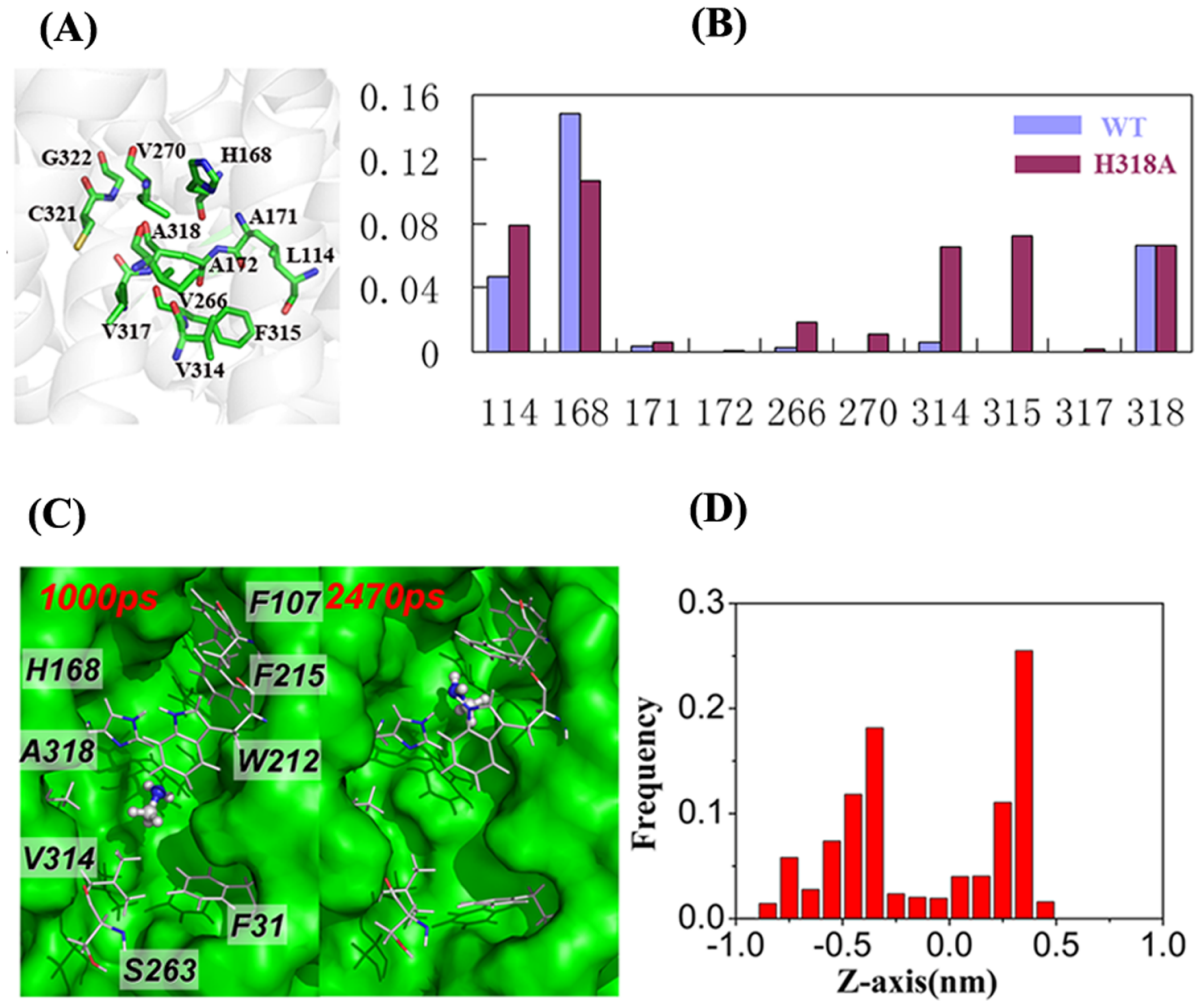
Substrate conduction through H318A AmtB. *A.*Neighboring residues around Ala318 in crystal structure 2NPE. *B*. The frequency of hydrophobic interaction pair between methylamine and the residues round residue 318 in the process of methylamine transport in the wild type (cyan) and H318A mutated EcAmtB (purple). Data were collected from trajectories A1 and C1, respectively. *C*, Two snapshots showing the most frequent staying locations of methylamine in the trajectory through H318A AmtB. *D*, Frequency distribution for the location of methylamine along the Z-axis of the channel in H318A AmtB (trajectory C1, data were collected in the whole trajectory).

Methylamine was trapped in two sites at z≈−0.48 nm and 0.30 nm in trajectory C1 (Figures 6C & D). In order to examine the reason for methylamine staying around these two sites, the structure of the His318A variant was examined, revealing that almost all the residues in the vicinity of residue 318 (z≈−0.5 nm) are hydrophobic, e.g. I114, I266 and V314 and F315. The frequency of the hydrophobic interactions between methylamine and residues around residue 318 was then calculated for both wild-type and the H318A EcAmtB variant (Figure 6B). It is clear that methylamine formed contacts with I114, I266, V314 and F315 around the site of z≈−0.5 nm more frequently in the H318A variant than in wild-type EcAmtB. Therefore the hydrophobic interactions between these residues and methylamine could be the main reason for the trapping of methylamine.

The energy barrier is about 2.51 kcal/mol from site Am4 (z≈−0.32 nm) to the cytoplasm (z≈−1.50 nm) for H318A variant conducting ammonia (Figure 4B). However, for methylamine, the energy barrier from site Am4 to the exit of the channel is about 6.22 kcal/mol, which is about 2.18 kcal/mol greater than that in the wild-type (Figure 4A). Furthermore, similar to the H168A variant, the H318A variant shows a deep potential energy well for methylamine (Figure 4A) which is 8.60 kcal/mol and 6.22 kcal/mol lower than the entrance and exit sites respectively, indicating that methylamine is also likely to be trapped in the region from z≈1.0 nm to −1.0 nm once it arrives the site Am2 (z≈0.0 nm). Within this region, two local energy minima are present (−3.67 kcal/mol at z≈−0.38 nm and −4.60 kcal/mol at z≈0.25 nm), corresponding to the positions at which trapping of the methylamine molecule was observed in the MD simulation (Figure 6D). Considering both the higher energy barrier for exit and the deep potential energy well for trapping the substrate inside the channel of the variant protein, the conduction of methylamine is expected to be impaired.

### Substrate Conduction through the H168A/H318A Variant of EcAmtB

In contrast to wild-type EcAmtB, the H168A/H318A variant neither transported methylamine nor supported growth on 3 mM NH_4_Cl. Surprisingly, our MD simulations showed that the substrates could exit to periplasm. To validate this result, more MD simulations were performed to investigate the mechanism underlying the dysfunction of this variant protein (trajectories D1 to D4, Table 2) with the two substrates initially located in the Am2 site in all cases. Interestingly, the simulation results showed that the substrate could not only exit the channel through the periplasmic vestibule but also pass through the channel and exit to the cytoplasm (see Figures 3D1 to 3D4). Members of the Amt protein family have been considered to function unidirectionally at low extracellular ammonium concentrations so as to conduct ammonia into the cell from the external medium. [19] Consequently the loss of the unidirectional property will result in the failure to conduct ammonia into the cell and the inability to support growth at low ammonium concentrations. Previous studies showed that the unidirectionality of the EcAmtB could be influenced by several factors, e.g., the concentration of magnesium ions or polyamines inside the cell. [66], [67] Our present studies showed that the dynamic function of AmtB protein could also affect the unidirectionality property.

Based on the abovementioned results, we speculate that the dysfunction of this H168A/H318A channel is likely to reflect the loss of the function of entrance gate formed by the conserved residues F107 and F215 (Figure 1C), which leads to the loss of the unidirectional conduction property of the channel. Indeed, our *in vivo* results showed that the F215A variant is inactive not only in methylamine transport assays but also in the ammonium growth assay (Figure 2A and Table 3), [23] confirming the important role of this residue in EcAmtB function. On the other hand, previous studies showed that the F107A variant has a similar function to wild-type EcAmtB. [23], [68] Moreover, our MD trajectories for wild-type and various variants also revealed that F107 is intrinsically flexible and not essential for substrate transport. [35] Therefore F215, but not F107, should play an essential role as a valve in the conduction channel.

**Figure 7:**
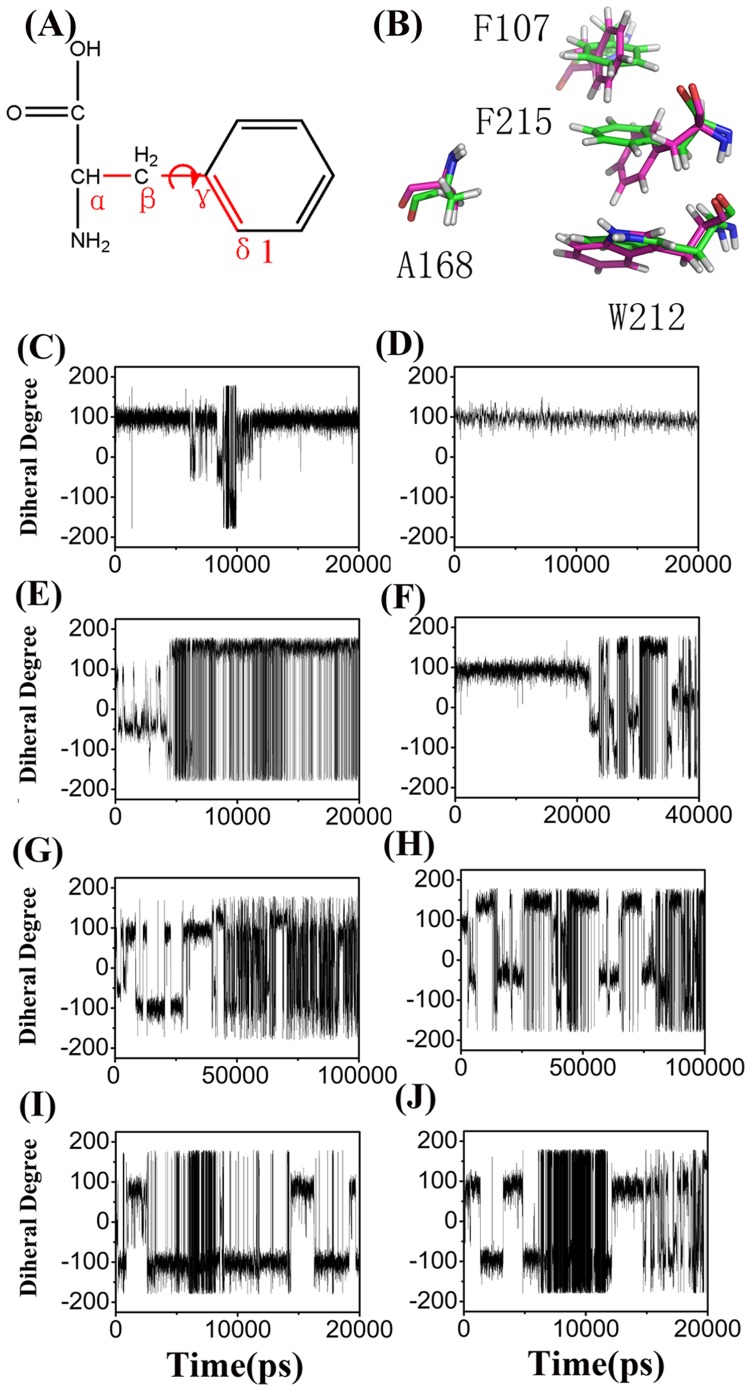
Analysis of the dynamics of the Phe gate. *A*, The torsion angle (C_α_-C_β_-C_γ_-C_δ1_ ) used for the analysis of F215 gating. *B*, The partially-stacked phenyl rings of F107 and F215 in the open (red) and closed (green) states (two snapshots are shown). *C-J*, The torsion angle C_α_-C_β_-C_γ_-C_δ1_ of F215 as a function of simulation time in the processes of substrate leaving the channel. Data were collected from trajectories of wild-type EcAmtB with methylamine (*C*) and ammonia (*D*), H168A/H318A AmtB with methylamine (*E, G*), ammonia (*F, H*), and H168A/H318A AmtB without substrate (*I, J*).

It was recently suggested that the torsion angle of C_α_-C_β_-C_γ_-C_δ1_ in F215 could be used to define the open state with a value of around 20° (±17°) or −166° (±14°) and closed state with a value of around 80°(±16°) or −100° (±15°) (Figures 7A & 7B). [32] In our MD simulations (trajectories D1–D6, Table 2), the torsion angle in the H168A/H318A EcAmtB (Figures 7E–J) were dramatically more dynamic than those in wild-type EcAmtB (Figures 7C & 7D), no matter whether there is a substrate inside the channel (Figures 7E–H) or not (Figures 7I–J), indicating that the gate in the H168A/H318A channel opened much more often than that in wild-type. Hence there is a significant chance of the substrate returning to the periplasmic side of the H168A/H318A channel. In summary, the change of inherent motion of the channel entrance gate caused by the double histidine mutation is likely to be the main reason for the dysfunction of H168A/H318A EcAmtB, suggesting that the failure of this variant to conduct MA has a different mechanism from that in the single His variants.

The PMF profile (Figure 4B) showed that for the H168A/H318A variant the energy barriers for ammonia (in the pore of channel) moving out of the conduction channel to periplasm (from z≈0.0 nm to 0.5 nm) or to cytoplasm (from z≈−0.50 nm to −1.0 nm) are 3.22 or 2.00 kcal/mol, respectively. The little difference in the barriers indicated the possibility of losing unidirectional conduction of the channel. Moreover, the energy barriers for ammonia passing the entrance gate to move into (from z≈1 nm to 0.5 nm) the conduction channel is 3.64 kcal/mol, even 0.42 kcal/mol larger than the barrier for ammonia out of the channel (from z≈0.0 nm to 0.5 nm), also suggesting that ammonia could be easily transported back to the periplasmic side. In addition, the PMF peak around the cytoplasmic exit (z≈−1.0 nm) is 3.10 kcal/mol, only 0.52 kcal/mol lower than the peak around the entrance (3.62 kcal/mol at z≈0.58 nm), indicating again the possibility of losing unidirectional conduction of the channel.

Similar to ammonia, all energy barriers for methylamine in the pore to pass the entrance and exit of the H168A/H318A variant (Figure 4A) are lower than those for methylamine in any other variant. The PMF profile (Figure 4A) showed that for the H168A/H318A variant, the energy barriers for methylamine (in the pore of channel) moving out of the conduction channel to the periplasm (from z≈0.0 nm to 0.5 nm) or to the cytoplasm (from z≈−0.50 nm to −1.0 nm) are 5.44 or 3.40 kcal/mol, respectively. The difference of 1.96 kcal/mol also indicated the possibility of losing unidirectional conduction of the channel. Moreover, the energy barrier for methylamine passing the gate of the H168A/H318A variant (from z≈1 nm to 0.5 nm) is about 3.78 kcal/mol, which is only 1.66 kcal/mol lower than the energy barrier for methylamine moving back to the periplasmic side (from z≈0.0 nm to z≈1.0 nm). In addition, the PMF peak around the entrance (z≈0.61 nm) is 4.34 kcal/mol, only 1.66 kcal/mol larger than the peak around the cytoplasmic exit (2.68 kcal/mol at z≈−0.94 nm). By contrast, the equivalent difference for the wild type is 2.55 kcal/mol. All these data suggest that, like ammonia, methylamine could also move in either direction in the H168A/H318A channel, resulting in the loss of unidirectional conduction. This is in agreement with our observations in MD simulations, namely that both substrates left the channel from both the periplasmic and cytoplasmic sides (Figures 3D1 to 3D4).

**Figure 8:**
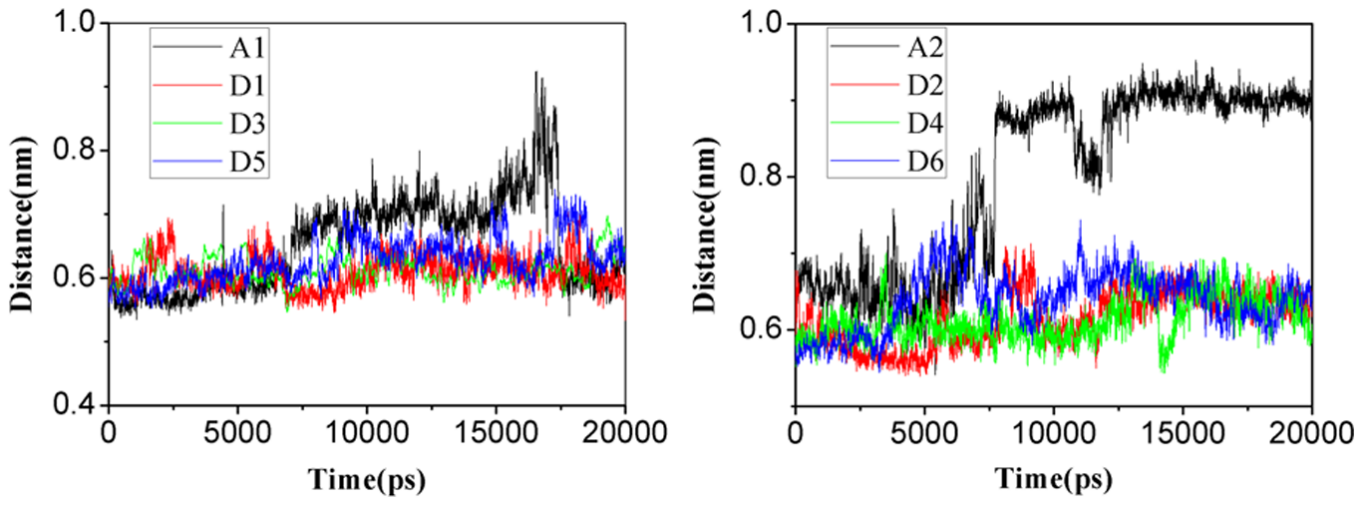
Distance between the sidechains of residues Phe31 and Val314 versus time in the process of substrate leaving the conduction channel (Only the first 20 ns were plotted for comparison). *A*. Simulations for MA. *B*. Simulations for NH_3_ in wild type AmtB (trajectories A1 and A2) and H168A/H318A variant (trajectories D1 to D6). A1 and A2 are colored in black, D1 and D2 are in red, D3 and D4 are in green, D5 and D6 are in blue, respectively.

During the simulation time, it was observed that water molecules could move in and out of the pore past the exit constriction formed by residues V314 and F31 in the wild type, which is consistent with our previous results. [33], [36] Water molecules were also frequently observed to stay around site Am4 (Figure S4), consistent with the crystal structure obtained by Zheng et al. [12] Moreover, water molecules could form hydrogen bonds with the substrate and thereby facilitate the substrate to move past the constriction of wild-type channel. [25], [36], [43] By contrast, few water molecules were observed to move and stay in the pore of the channel in the H168A/H318A variant during our MD simulations (trajectories D1 to D6, Table 2). By analyzing the trajectories carefully, we found that the distance between the residues V314 and F31 in the H168A/H318A variant is apparently shorter than that in the wild-type (Figure 8). So the constriction formed by these two hydrophobic residues might prevent water molecules moving into the pore in the H168A/H318A variant, impairing its performance in conducting substrates.

## Discussion

In the present study, we used a combination of *in vivo* experiments and molecular dynamics simulations to investigate the function of the conserved histidine residues in the pore of ammonia transport proteins, and the different behaviors of ammonia and methylamine as channel substrates. Both the *in vivo* experiments and the simulations showed that although changing either of the conserved EcAmtB histidine residues (His168 or His318) to alanine results in the failure to transport methylamine, these single His variants still support growth on ammonia. However, the double histidine variant (H168A/H318A) loses its ability to transport both methylamine and ammonia. The same phenotypes were found for a comparable set of single and double His variants in ScMep2.

The ability of some His variants to transport ammonia has been observed previously. Hall and Kustu [68] showed that EcAmtB H168E, H168D and H318D were all effective in supporting growth on low ammonium, and Rutherford et al. [69] also reported that ScMep2 H348A was competent to grow on low ammonium. Nevertheless both His residues have been strongly conserved during evolution, and the only common natural variant is a glutamate substitution of the first His in a number of fungal Amt proteins including ScMep1 and ScMep3. [44] Boeckstaens et al. [70] reported that a ScMep2 H194E variant was still competent to transport ammonium and methylammonium but had an altered pH optimum for transport. However whether this specific substitution in fungal Amt proteins has a particular biochemical or physiological function is presently unclear.

Disparities between the data obtained when using either methylamine uptake assays or growth on low levels of ammonium were also observed recently by Hall et al. [68] They reported that trends in methylamine transport and growth on low ammonium did not always parallel one another, and that methylamine exhibited more variability in reporting AmtB function relative to the growth assay on low ammonium. They concluded that, for reasons yet to be determined, AmtB handles ammonium and MA differently. [68] Our MD studies on EcAmtB offer an explanation for this in the relative size and hydrophobicity of the two substrates and their interactions with residues within the channel. Furthermore we have shown that these characteristics may alter if residues within the channel are changed.

The present simulation results showed that although the overall structures of the variant channels are very similar to the wild-type, when the polar residue histidine is mutated to the hydrophobic residue alanine, the hydrophobicity of the channel pore in each of the three mutants (H168A, H318A, and H168A/H318A) becomes higher than that of the wild-type. In addition, the PMF calculations revealed that, compared to wild-type EcAmtB, the single histidine variants lead to deeper and negative potential energy wells for methylamine but not for ammonia passing through the conduction channel. It is then reasonable to speculate that the more hydrophobic environment in the mutant channel exerts more constraints on the hydrophobic methylamine molecule and results in a dysfunction in conducting methylamine. By contrast, inorganic ammonia is barely influenced by the changed environment in the conduction channel due to the change of a single histidine. The double histidine variant loses its function to transport either methylamine or ammonia into the cell by losing its unidirectional property as this variant changes the intrinsic dynamic states of the gate formed by F215 and lowers the energy barrier to the periplasmic side.

These observations provide new insights into the roles of the two conserved histidine residues in Amt proteins. Whilst it is apparent from both *in vivo* data and MD simulations that both His residues are not absolutely required for ammonia conduction, the MD data confirm that two His residues are required for optimum functionality thereby explaining why their conservation has been strongly selected. Our studies also highlight the potential problems associated with using methylamine uptake as the sole method to evaluate the function of AmtB derivatives, because whereas MA appears to be a reasonable substitute for NH_3_ in studying wild-type EcAmtB it does not mimic NH_3_ in many variants. This may also explain our previous failure to detect transport of [^14^C] MA by the *N. europaea* Rhesus (Rh50) protein despite its ability to support ammonium-dependent growth when expressed in a *S. cerevisiae Δmep* strain. [47].

## Supporting Information

Figure S1 **Thermodynamic cycles for the methylamine and ammonia perturbations in water (A) and at the site Am2 of H168A variant (B).** The unit is kcal/mol.
(TIF)

Figure S2 **The residues of wild type EcAmtB involved in hydrogen bonds and hydrophobic interactions with the substrate (CH_3_NH_2_ or NH_3_) versus simulation time in the trajectories A1 and A2.** (A) Time-dependent hydrophobic interactions between methylamine and the residues in the channel in trajectory A1. The important residues involved with the interactions are listed with colors. (B) Time-dependent hydrogen bonds formed between methylamine and the residues in the channel in trajectory A1. (C) Time-dependent hydrogen bonds formed between ammonia and the residues in the channel in trajectory A2.
(TIF)

Figure S3 **PMFs with error bar present for substrate permeation across wild-type and His variants of AmtB.** Permeation of substrate across H168A (A), H318A (B), wild-type (C) and H168A/H318A (D) AmtB. The data for CH_3_NH_2_ and NH_3_ are colored by black and red respectively.
(TIF)

Figure S4 **Exit of an ammonia molecule from the channel with the help of water molecules by forming hydrogen bond around the exit gate, and the process of water molecules entering and exciting the hydrophobic channel in trajectory A2.** Eight snapshot structures (1786, 1788, 1790, 2684, 2686, 2688, 2696 and 2712 ps) are displayed.
(TIF)

Text S1 **Free-energy perturbation.**
(DOC)
