# Supplementary figures and images for: Ammonium Transport Proteins with Changes in One of the Conserved Pore Histidines Have Different Performance in Ammonia and Methylamine Conduction
Source: PLoS One. 2013 May 7;8(5):e62745. doi: 10.1371/journal.pone.0062745 (PMC3647058; doi:10.1371/journal.pone.0062745)

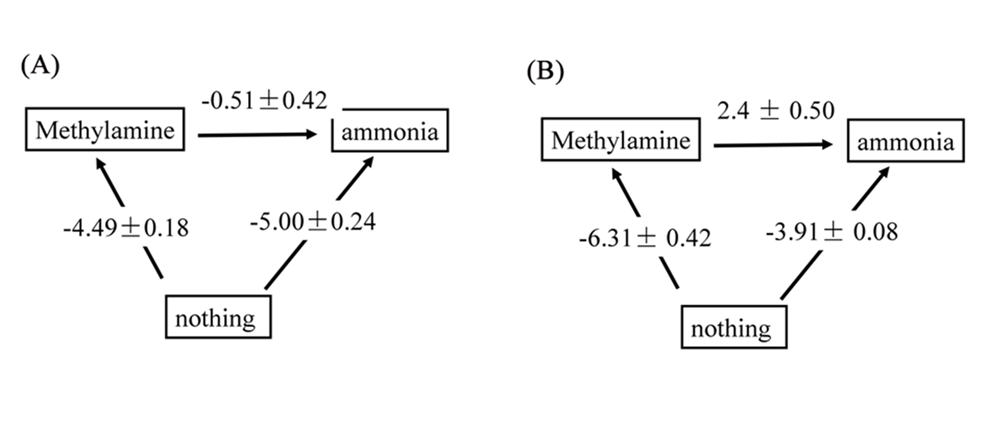

Supplement: Figure S1 — Thermodynamic cycles for the methylamine and ammonia perturbations in water (A) and at the site Am2 of H168A variant (B). The unit is kcal/mol. (TIF) [file pone.0062745.s001.tif]

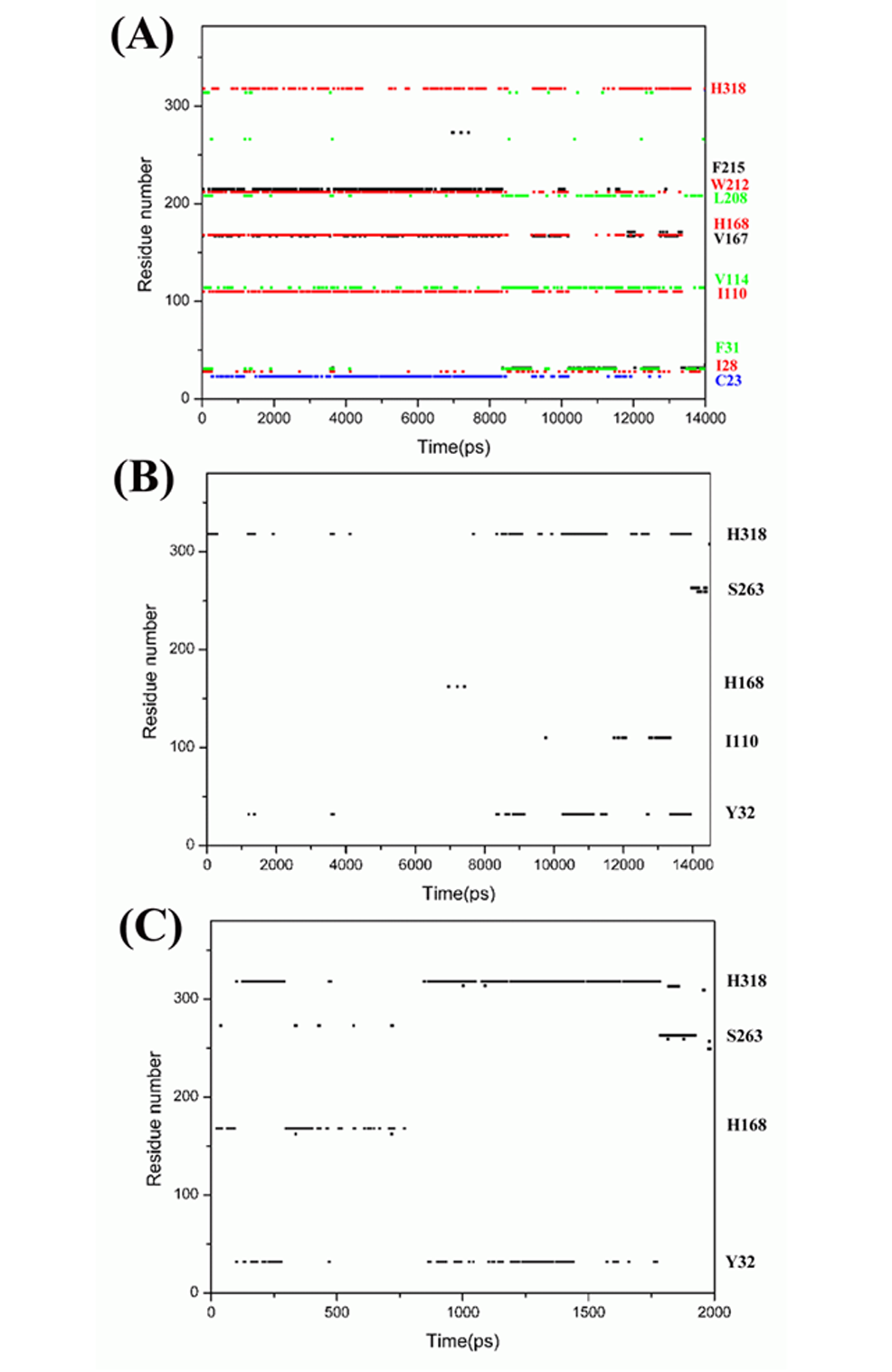

Supplement: Figure S2 — The residues of wild type EcAmtB involved in hydrogen bonds and hydrophobic interactions with the substrate (CH3NH2 or NH3) versus simulation time in the trajectories A1 and A2. (A) Time-dependent hydrophobic interactions between methylamine and the residues in the channel in trajectory A1. The important residues involved with the interactions are listed with colors. (B) Time-dependent hydrogen bonds formed between methylamine and the residues in the channel in trajectory A1. (C) Time-dependent hydrogen bonds formed between ammonia and the residues in the channel in trajectory A2. (TIF) [file pone.0062745.s002.tif]

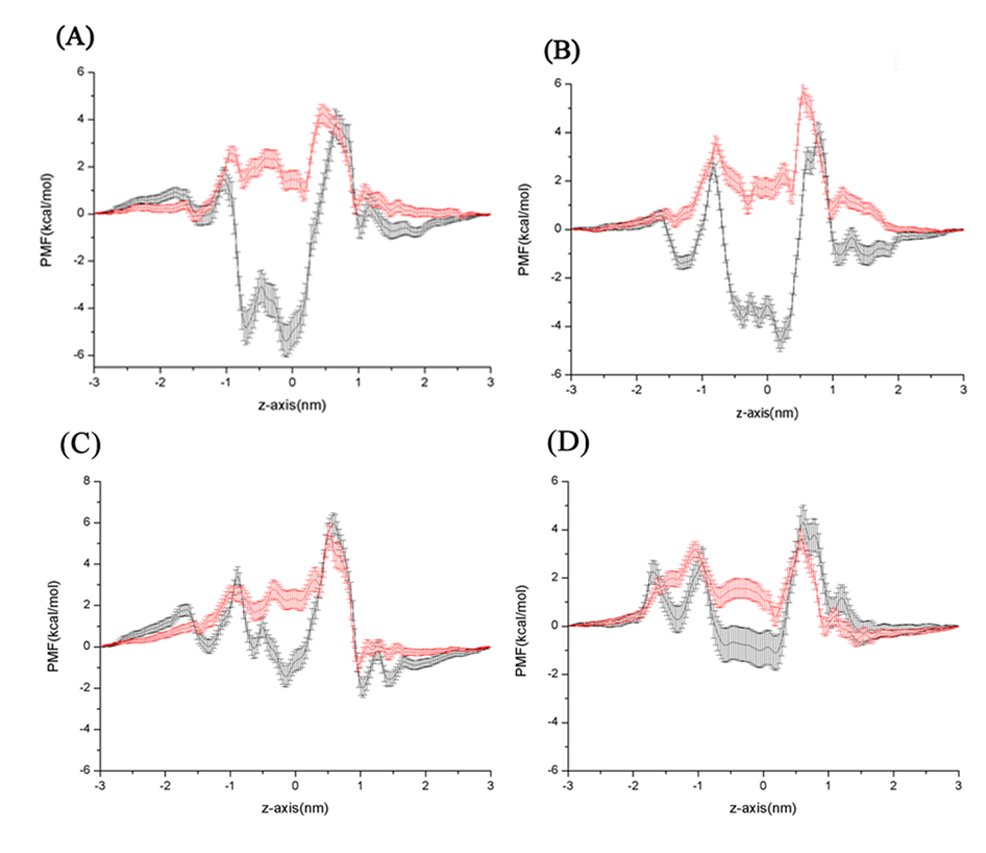

Supplement: Figure S3 — PMFs with error bar present for substrate permeation across wild-type and His variants of AmtB. Permeation of substrate across H168A (A), H318A (B), wild-type (C) and H168A/H318A (D) AmtB. The data for CH3NH2 and NH3 are colored by black and red respectively. (TIF) [file pone.0062745.s003.tif]

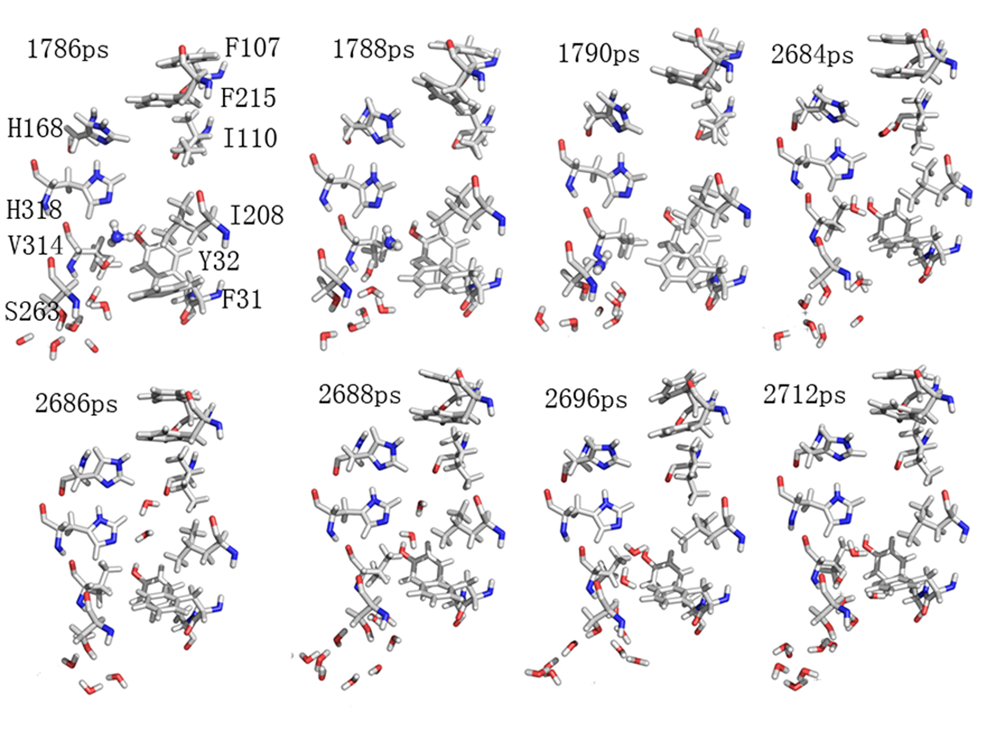

Supplement: Figure S4 — Exit of an ammonia molecule from the channel with the help of water molecules by forming hydrogen bond around the exit gate, and the process of water molecules entering and exciting the hydrophobic channel in trajectory A2. Eight snapshot structures (1786, 1788, 1790, 2684, 2686, 2688, 2696 and 2712 ps) are displayed. (TIF) [file pone.0062745.s004.tif]
