# Supplementary material for: Ammonium Transport Proteins with Changes in One of the Conserved Pore Histidines Have Different Performance in Ammonia and Methylamine Conduction
Source: PLoS One. 2013 May 7;8(5):e62745. doi: 10.1371/journal.pone.0062745 (PMC3647058; doi:10.1371/journal.pone.0062745)
Supplement: Text S1 — Free-energy perturbation. (DOC) [file pone.0062745.s005.doc]

Supporting Information

Ammonium Transport Proteins with Changes in One of the Conserved Pore Histidines Have Different Performance in Ammonia and Methylamine Conduction

Jinan Wang ^1§^, Tim Fulford ^2§^, Qiang Shao^1^, Arnaud Javelle^2†^, Huaiyu Yang^1^, Weiliang Zhu^1*^ and Mike Merrick^2*^

^1^Drug Discovery and Design Center, CAS Key Laboratory of Receptor Research, Shanghai Institute of Materia Medica, Chinese Academy of Sciences, Shanghai, China

^2^Department of Molecular Microbiology, John Innes Centre, Norwich Research Park,

Norwich, United Kingdom

^†^ Current address: Division of Molecular Microbiology, College of Life Sciences, University of Dundee, Dundee, United Kingdom

^§^Authors contributed equally to this work

^*^To whom correspondence should be addressed: Mike Merrick, Tel: +44 1603 450749, Fax: +441603 450778, E-mail: [mike.merrick@jic.ac.uk](mailto:mike.merrick@jic.ac.uk); Weiliang Zhu, Tel: +86 21 50805020, Fax: +86 21 50807088, E-mail: wlzhu@mail.shcnc.ac.cn.

**Text S1 Free-energy perturbation**

The solvation free energies (ΔG_solv_) or the relative binding free energy difference between methylamine and ammonia (ΔG(MA 🡪 NH_3_)) in water or at site Am2 of H168A variant were calculated using the free-energy perturbation method described by Shirts et al (see the thermodynamic cycles in Figure S1).[1] Simulations were performed separately at 21 different alchemical intermediate λ values: 0, 0.05, 0.1, 0.15, 0.20, 0.25, 0.30, 0.35, 0.40, 0.45, 0.50, 0.55, 0.60, 0.65, 0.70, 0.75, 0.80, 0.85, 0.90, 0.95 and 1. In these simulations, the coupled state (λ=1) corresponds to a simulation where the solute (ammonia or methylamine) is fully interacting with the environment and the uncoupled state (λ=0) corresponds to a simulation where the solute does not interact with the environment. Each window corresponds to an independent simulation that includes 150 ps of equilibration and subsequent 350 ps of data collection. Hydration free energies were computed using the Bennett acceptance ratio (BAR).[2] The simulation temperature was kept constant at 300 K by coupling the system to a Nose´-Hoover thermostat (τ = 0.5 ps). [3,4] And, the pressure was kept at 1 bar using the Parrinello- Rahman[5]pressure coupling scheme (τ = 1 ps). The cutoff for Lennard-Jones interaction was set as 10 Å.

**References:**

1. Shirts MR, Pitera JW, Swope WC, Pande VS (2003) Extremely precise free energy calculations of amino acid side chain analogs: Comparison of common molecular mechanics force fields for proteins. J. Chem. Phys. 119: 5740-5761.

2. Bennett CH (1976) Efficient estimation of free energy differences from Monte Carlo data. Journal of Computational Physics 22: 245-268.

3. Hoover WG (1985) Canonical dynamics: Equilibrium phase-space distributions. Phys Rev A 31: 1695-1697.

4. Nose´ S (1984) A molecular dynamics method for simulations in the canonical ensemble. Mol Phys 52: 255-268.

5. Parrinello M, Rahman A (1981) Polymorphic transitions in single crystals: A new molecular dynamics method. J Appl Phys 52: 7182-7190.
